# Maternal BMI, early-life growth, and atopic dermatitis by age 3 years

**DOI:** 10.1016/j.jacig.2026.100693

**Published:** 2026-03-23

**Authors:** Frida Victoria Berents, Marius Kurås Skram, Karin C. Lødrup Carlsen, Berit Granum, Guttorm Haugen, Gunilla Hedlin, Christine Monceyron Jonassen, Björn Nordlund, Andrew Reiner, Håvard Ove Skjerven, Anne Cathrine Staff, Cecilie Svanes, Riyas Vettukattil, Cilla Söderhäll, Eva Maria Rehbinder

**Affiliations:** a Faculty of Medicine, University of Bergen, Bergen, Norway; b Faculty of Medicine, Institute of Clinical Medicine, University of Oslo, Oslo, Norway; c Division of Paediatric and Adolescent Medicine, Oslo University Hospital, Oslo, Norway; f Division of Obstetrics and Gynaecology, Oslo University Hospital, Oslo, Norway; k Oslo Centre for Biostatistics and Epidemiology, Oslo University Hospital, Oslo, Norway; n Department of Dermatology, Oslo University Hospital, Oslo, Norway; d Department of Specialist Professional Development, the Directorate of Health, Oslo, Norway; e Department of Chemical Toxicology, Norwegian Institute of Public Health, Oslo, Norway; j Department of Virology, Norwegian Institute of Public Health, Oslo, Norway; g Department of Women's and Children's Health, Karolinska Institutet, Stockholm, Sweden; h Astrid Lindgren Children's Hospital, Karolinska University Hospital, Stockholm, Sweden; i Center for Laboratory Medicine, Østfold Hospital Trust, Kalnes, Norway; l Centre of International Health, Department of Global Public Health and Primary Care, University of Bergen, Bergen, Norway; m Department of Occupational Medicine, Haukeland University Hospital, Bergen, Norway

**Keywords:** PreventADALL, anthropometrics, fetal growth, BMI, atopic dermatitis

## Abstract

**Background:**

Childhood atopic dermatitis is associated with maternal gestational weight gain, whereas an association with prepregnancy body mass index (ppBMI) or fetal and newborn anthropometric measurements is unclear.

**Objective:**

Our aim was to determine whether, primarily, maternal ppBMI and, secondarily, offspring newborn anthropometric measurements or fetal growth are associated with atopic dermatitis by age 3 years.

**Methods:**

In 2107 mother-child pairs (54% boys) from the general population–based Scandinavian PreventADALL (Preventing Atopic Dermatitis and Allergies) cohort, maternal ppBMI was reported at time of enrollment (at midpregnancy). Thoracic and abdominal circumferences were measured by ultrasound at midpregnancy and again at birth, as were weight and length. Fetal growth, including thoracic and abdominal growth and fetal weight gain, was estimated from midpregnancy to birth. Atopic dermatitis was diagnosed using standard criteria at 3, 6, 12, 24, and age 36 months.

**Results:**

Atopic dermatitis was diagnosed in 525 of the 2107 children (25%) by age 3 years, with a positive association with increasing maternal BMI (adjusted odds ratio [aOR] per BMI unit = 1.03 [95% CI = 1.00-1.06]). Furthermore, increasing birth length (mean = 50.5 ± 2.0 cm) was positively associated with atopic dermatitis (aOR = 1.06 [95% CI = 1.01-1.12]), whereas short birth length (<48 cm) was inversely associated with atopic dermatitis (aOR = 0.71 [95% CI = 0.51-1.0]). Neither birth weight nor thoracic, abdominal, or upper arm circumference at birth nor fetal growth was associated with atopic dermatitis.

**Conclusion:**

Increasing maternal ppBMI and increasing birth length were positively associated with offspring atopic dermatitis by age 3 years, whereas short birth length was associated with a lower risk of atopic dermatitis.

Atopic dermatitis (AD) is the most common skin disease in children, typically developing during the first years of life. The condition is characterized by inflammation and impaired skin barrier function, presenting as eczema with dry and itchy skin.^1^ Most cases occur before age 5 years, and the disease can persist into adolescence and adulthood.

The most significant risk factor for the development of AD is a positive family history of atopic disease, as well as presence of other atopic conditions in the child.^2^^,^^3^ Mutation in the filaggrin gene, another major risk factor, is present in 30% to 50% of patients with AD versus in 5% to 10% of the general population.^2^ Filaggrin is an essential epidermal protein, and mutations in this gene lead to impaired skin barrier function, which manifests clinically as dry and flaky skin.^4^

The proportion of women entering pregnancy with a higher body mass index (BMI) is increasing.^5^ Overweight or obesity in adults older than 18 years has increased worldwide from 25% in 1990 to 43% in 2022.^6^ Although a Chinese study found that maternal prepregnancy overweight or obesity was associated with a 20% increase in the odds of infant AD,^7^ this finding was not supported by a prospective cohort study of 13,269 children aged 10 to 17 years from the United States.^8^ However, the US study found that maternal gestational weight gain (GWG) of 15.9 to 20 kg was associated with 11% increased odds and GWG of 20.4 kg or more was associated with 23% increased odds of development of AD.^8^ Additionally, a systematic review and meta-analysis reported that high maternal GWG increased the risk of childhood AD, whereas low maternal GWG decreased the risk.^9^

In the prospective German Ulm SPATZ Health Birth Cohort Study from 2012, which included 1006 children, fetal measurements were associated with AD, including low and high second-trimester abdominal circumference, a high second-trimester ratio of head circumference to abdominal circumference ratio, and faltering second- to third-trimester head circumference.^10^ Conversely, a higher third-trimester femur length was associated with decreased risk of childhood AD.^10^ Furthermore, a longitudinal UK birth cohort study tracking fetal measurements in the first and second trimester demonstrated that increased birth weight was associated with a higher risk of AD.^11^ Although maternal GWG has been repeatedly associated with childhood AD, whether maternal prepregnancy BMI (ppBMI) or fetal and newborn anthropometric measurements are associated with offspring AD remains unclear.^8^

The aims of this study were therefore to explore whether (1) maternal ppBMI, (2) newborn anthropometric measurements, and/or (3) fetal growth were associated with development of AD by age 3 years.

## Methods

### Study design

Preventing Atopic Dermatitis and Allergy in Children (PreventADALL) is a Nordic multicenter, nonselected birth cohort study with 2 main aims: (1) assess whether atopic diseases could be prevented through early infant skin or food interventions and (2) explore early-life factors that may be implicated in the development of noncommunicable disease. The present study has been conducted within the exploratory part of the PreventADALL study, which is being conducted in Norway at Oslo University Hospital and the Østfold Hospital Trust, Kalnes, and in Sweden at Karolinska University Hospital, Stockholm. Pregnant women were enrolled at the 18-week routine ultrasound examination.^12^ The exclusion criteria were pregnancies with more than 2 fetuses; insufficient proficiency in a Scandinavian language; plans to move outside a reasonable travel distance within 1 year after delivery; fetuses with severe malformations or disease; and presence of severe maternal, fetal, or neonatal disease.^13^ Infants born from gestational week 35 onward without any serious illnesses were included at birth. The children attended follow-up visits including clinical examinations at ages 3, 6, and 12 months, as well as at ages 2 and 3 years. Electronic questionnaires including health, demographic, and socioeconomic factors were completed by the mother at enrollment; at week 34 of pregnancy; and at ages 3, 6, 9, 12, 18, 24, 30, and 36 months.

Written informed consent was obtained from the mothers at enrollment and from both parents at inclusion of the newborn. The PreventADALL study was approved by the Regional Committee for Medical and Health Research Ethics in South-Eastern Norway (approval no 2014/518) and in Sweden (approval no 2014/2242-31/4), and it was registered at ClinicalTrials.gov (identifier NCT02449850).

### Study population

**Fig 1:**
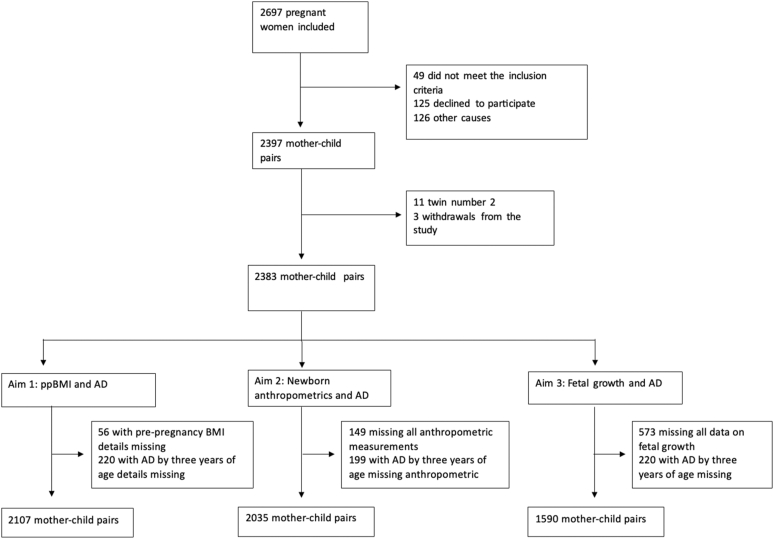
Flowchart showing the selection process of the study population from the PreventADALL cohort. The numbers of excluded participants and reasons for exclusion are shown, as are the final numbers of mother-child pairs included in the analysis for aim 1 (investigating the association between ppBMI and offspring AD by age 3 years [n = 2107]), aim 2 (investigating the association between newborn anthropometrics and offspring AD by age 3 years [n = 2035]), and aim 3 (investigating the association between fetal growth and offspring AD by age 3 years [n = 1590]).

From December 2014 to October 2016, a total of 2697 pregnant women were recruited for the PreventADALL study in Oslo, Østfold, and Stockholm.^14^ In the present study, the second-born twin of 11 twin pairs was excluded. We included all children with available information on AD diagnosis. The study population for aim 1 (association between ppBMI and AD) included 2107 mother-child pairs with complete data on exposure and outcome. For aim 2 (newborn anthropometric measurements), 2035 mother-child pairs were included, and for aim 3 (fetal growth anthropometrics), 1590 mother-child pairs were included (Fig 1).

### Exposures

ppBMI was divided into 4 categories by using the World Health Organization classification (Table I).^15^ Fetal midpregnancy anthropometric measurements included thoracic and abdominal circumference as well as estimated fetal weight (EFW) calculated using the Combs formula, which is a regression-based equation used in obstetric ultrasound to estimate fetal weight from biometric measurements (Table I).^16^ In addition, birth weight and length, as well as newborn (within 2 days after birth) thoracic, abdominal, and left upper arm circumference, were measured and used within the present study population both as continuous variables and percentiles (either above the 90th or below the 10th percentile) (Table I). The standard operating procedures for the anthropometric measurements can be found in the Supplementary Material (see the Online Repository at www.jaci-global.org). Newborns missing all of the anthropometric measurements were excluded.

**Table I:** Overview of all anthropometric exposure variables in the present study

Exposure	Continuous	Categoric
Maternal		
ppBMI	ppBMI (kg/m^2^)	Underweight (<18.5 kg/m^2^) Normal weight (18.5-24.9 kg/m^2^) Overweight (25.0-29.9 kg/m^2^) Obese (>30 kg/m^2^)
Birth/newborn anthropometric measurements		
Birth length	Birth length (cm)	Long birth length is defined as >90th percentile, >53 cm
		Short birth length is defined as <10th percentile, <48 cm
Birth weight	Birth weight (g)	High birth weight is defined as >90th percentile, >4180 g
		Low birth weight is defined as <10th percentile, <2994 g
Thoracic circumference	Thoracic circumference (cm). The mean of 3 measurements is used	Large thoracic circumference is defined as >90th percentile, >36.4 cm
		Small thoracic circumference is defined as <10th percentile, <31.7 cm
Abdominal circumference	Abdominal circumference (cm). The mean of 3 measurements is used	Large abdominal circumference is defined as >90th percentile, >35.5 cm
		Small abdominal circumference is defined as <10th percentile, <30.0 cm
Upper arm circumference	Upper arm circumference (cm). The mean of 2 measurements is used	Large upper arm circumference is defined as >90th percentile, >12.4 cm
		Small upper arm circumference is defined as <10th percentile, <10.0 cm
Fetal growth based on fetal and birth anthropometric measurements		
Fetal weight gain Fetal weight, calculated by the Combs formula as follows: *EFW* = (0.23718 × *AC2* × *FL*) + (0.03312 × *HC3*), where *AC* is abdominal circumference, *FL* is femur length, and *HC* is head circumference^16^	Fetal weight gain (g/d): calculated as fetal weight gain, as weight increase (g/d) from the midpregnancy ultrasound examination until birth (newborn)	High fetal weight gain is defined as >90th percentile, >14.64 g
		Low fetal weight gain is defined as <10thpercentile, <10.65 g
Fetal thoracic circumference growth	Fetal thoracic circumference growth (mm/d): calculated as fetal thoracic circumference growth (mm/d) from the midpregnancy ultrasound examination until birth (newborn). The mean of 3 measurements in the fetus is used	High fetal thoracic circumference growth is defined as >90th percentile, >0.94 mm/d
		Low fetal thoracic circumference growth is defined as <10th percentile, <0.75 mm/d
Fetal abdominal circumference growth	Fetal abdominal circumference growth (mm/d): calculated as fetal abdominal circumference growth (mm/d) from midpregnancy ultrasound examination until birth (newborn). The mean of 3 measurements in the fetus	High fetal abdominal circumference growth is defined as >90th percentile, >0.83 mm/d
		Low fetal abdominal circumference growth is defined as <10th percentile, <0.62 mm/d

A fetal ultrasound examination was performed around 18 weeks’ gestational age by trained midwives. At Karolinska University Hospital, only fetal head circumference and femur length were routinely recorded, whereas at Oslo University Hospital and the Østfold Hospital Trust, head circumference, abdominal circumference and femur length were part of the routine ultrasound, with thoracic circumference also measured in the women interested in joining the PreventADALL study. All measurements were obtained 3 times and averaged for the analyses. Thoracic circumference was measured in the axial plane at the level of the 4-chamber view of the heart, using an ellipse along the ribs.^17^ One fetal medicine obstetrician trained all midwives measuring thoracic circumference and ensured quality control through random assessment of measurement samples, thereby reducing the interoperator variability.^17^

Fetal growth was calculated as EFW gain (weight increase per day, as determined from ultrasound measurement from midpregnancy to birth), as well as from thoracic and abdominal circumference growth rate per day from midpregnancy ultrasound measurements until the newborn examination (Table I). Gestational age was calculated on the basis of femur length.

### Outcomes

The main outcome was AD by age 3 years, as diagnosed through a comprehensive clinical examination by trained study personnel applying validated diagnostic criteria, including the criteria of the UK Working Party (at all timepoints) or the Hanifin and Rajka criteria diagnostic criteria for AD (at ages 1, 2, and 3 years).^18^^,^^19^ AD was diagnosed by fulfilling either the UK Working Party or Hanifin and Rajka criteria at any timepoint by age 3 years.

### Statistical analysis

Categoric variables are presented as numbers and percentages, whereas continuous variables are reported as means, SDs, and minimum-to- maximum values. A significance level of .05 was used throughout the analyses. Differences in baseline characteristics between the study population and the rest of the PreventADALL cohort were assessed by using chi-square tests for categoric variables and Student *t* tests for continuous variables.

Risk of AD was analyzed by using univariable and multivariable logistic regression. The exposure variables were ppBMI, both as a continuous and a categoric variable, as well as continuous and categoric fetal and neonatal anthropometric measurements (Table I). Models were adjusted for relevant confounders. We performed complete-case analyses; participants with missing data on the exposure or outcome variables relevant to each aim were excluded. Consequently, the study population differed slightly depending on the aim and availability of ppBMI, anthropometric measurements and AD status by age 3 years (Fig 1).

Possible confounders were identified by using previous literature and selected using a directed acyclic graph (DAG) for each model.^20^ According to the specified causal model, maternal AD and maternal education were identified as confounders of the association between maternal ppBMI and offspring AD (see Fig E1 in the Online Repository at www.jaci-global.org), whereas for the association between newborn and fetal anthropometric measurements and the development of AD, we adjusted for maternal education and maternal ppBMI identified in the directed acyclic graphs (see Figs E2 and E3 in the Online Repository at www.jaci-global.org). As the interventions in the present study did not affect the outcome of AD by age 3 years,^12^^,^^21^ they were not considered relevant confounders in the present analysis.

The statistical analyses were conducted using Stata, version 17 (StataCorp, College Station, Tex).

## Results

Among the 2107 mother-child pairs (53% of which included boys), 25% (525 of 2107 children) were diagnosed with AD by age 3 years. A higher proportion of children with AD had mothers with a history of AD, asthma, or other atopic diseases, and filaggrin gene mutations were more common in the children who developed AD (Table II).

**Table II:** Baseline characteristics of the population of the present study, from the PreventADALL mother-child birth cohort comparing infants with and without AD by age 3 years

Characteristics	AD by age 3 y			*P* value
	No	Yes	Total	
Subjects, no. (%)	1582 (75)	525 (25)	2107 (100)	
Sex (n = 2107), no. (%)				.270
Female	749 (76)	234 (24)	983 (100)	
Male	833 (74)	291 (26)	1124 (100)	
Dwelling (n = 2107), no. (%)				.336
City	1104 (74)	378 (26)	1482 (100)	
Suburb/countryside	478 (76)	147 (24)	625 (100)	
Education (n = 1910), no. (%)				.324
High school or less, no. (%)	156 (78)	43 (22)	199 (100)	
<4 y of higher education	452 (76)	140 (24)	592 (100)	
≥4 y of higher education	829 (74)	290 (26)	1119 (100)	
Mother’s origin (n = 1919), no. (%)				.012
Scandinavia	1309 (75)	430 (25)	1739 (100)	
Rest of the world	134 (74)	46 (26)	180 (100)	
Mother diagnosed with AD (n = 1919), no. (%)				<.001
Yes	253 (66)	128 (34)	381 (100)	
No	1190 (77)	348 (23)	1538 (100)	
Mother diagnosed with asthma (n = 1919), no. (%)				<.001
Yes	205 (64)	117 (36)	322 (100)	
No	1238 (78)	359 (22)	1597 (100)	
Mother diagnosed with any atopic disease (n = 1919), no. (%)				<.001
Yes	542 (69)	248 (31)	790 (100)	
No	901 (80)	228 (20)	1129 (100)	
Filaggrin mutation (n = 2107), no. (%)				<.001
Yes	84 (56)	66 (44)	150 (100)	
No	1155 (77)	350 (23)	1505 (100)	
Unknown	343 (76)	109 (24)	452 (100)	
Birth mode (n = 2105), no. (%)				.203
Vaginal	1342 (76)	429 (24)	1771 (100)	
Caesarean section	238 (71)	96 (29)	334 (100)	
Age of mother (n = 2107) (y), mean ± SD	32.4 ± 4.2	32.7 ± 4.0	32.5 ± 4.1	.203
Gestational age at birth (wk) (n = 2102), mean ± SD	40.0 ± 9.6	40.2 ± 9.0	40.1 ± 9.4	.112
Birth weight (g) (n = 2100), mean ± SD	3565.3 ± 485.1	3598.3 ± 447.9	3573.5 ± 476.2	.169

The 3 most common loss-of-function mutations of the filaggrin gene in Europe (p.Arg501Ter, p.Ser761fs, and p.Arg2447Ter) were investigated. Having a filaggrin mutation (mutation status yes) was defined as being carrier of any of the 3 mutations.

### Maternal ppBMI and offspring AD development by age 3 years

**Fig 2:**
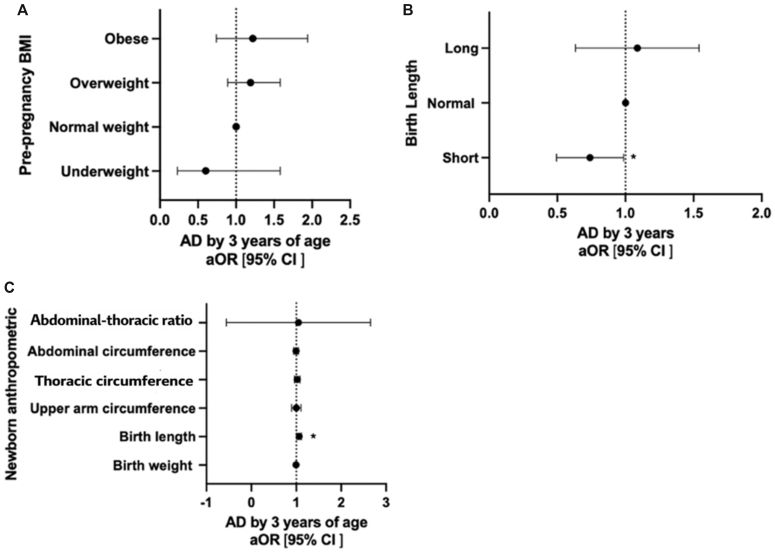
**A-C**, Forest plot showing the associations between ppBMI (n = 2107) (**A**), birth length (n = 1590) (**B**), and newborn anthropometric measurements (n = 2035) (**C**) and AD by age 3 years. Results are presented as odds ratios (ORs) with 95% CIs. ppBMI categories (ie, underweight [BMI < 18.5 kg/m^2^], normal weight [BMI = 18.5-24.9 kg/m^2^], overweight [BMI = 24.9-29.9 kg/m^2^], and obese [BMI > 30 kg/m^2^]) were based on the World Health Organizations definitions. All estimates were obtained from multivariable logistic regression models adjusted for maternal AD and maternal socioeconomic status, as identified by a directed acyclic graph. **C**, CIs are narrow for most anthropometric measurements, with the exception of abdominal-to-thoracic ratio.

Maternal ppBMI was within the normal range in 1637 children (78%), whereas 31 children (2%) were underweight, 326 (16%) were overweight, and 113 (5%) were obese. Increasing maternal ppBMI was positively associated with offspring AD by age 3 years in both the unadjusted and adjusted models (adjusted odds ratio [aOR] = 1.03 [95% CI = 1.00-1.06]; *P* = .043). This corresponds to a 3% increase in odds per 1-unit increase in maternal ppBMI (Table II and Fig 2, *A*). However, neither high nor low maternal ppBMI was associated with offspring AD when compared with normal weight (BMI = 18.5-24.9) (Table III).

**Table III:** Logistic regression of maternal ppBMI and the risk of AD in children by age 3 years unadjusted and adjusted for relevant confounders

Characteristics	Crude OR (95% CI)∗	*P* value∗	aOR (95% CI)†	*P* value†
Maternal ppBMI (kg/m^2^) (n = 2107), mean (range)	1.02 (0.99-1.04)	.224	1.03 (1.00-1.06)	.043
Underweight (BMI <18.5), mean (range)	0.58 (0.22-1.53)	.273	0.60 (0.23-1.58)	.296
Normal weight (BMI 18.5-24.9), reference value	1		1	
Overweight (BMI 25.0-29.9), mean (range)	1.14 (0.84-1.45)	.474	1.19 (0.89-1.58)	.242
Obese (BMI >30), mean (range)	0.95 (0.61-1.49)	.829	1.22 (0.74-1.94)	.453

Maternal ppBMI is a continuous variable; the rest of the variables are categoric.

*OR,* Odds ratio.

∗ ORs and *P* values from univariable logistic regression models, comparing the risk of AD by age 3 years with ppBMI.

† ORs and *P* values from multivariable logistic regression models, comparing the risk of AD by age 3 years of age with ppBMI, adjusted for maternal AD and maternal educational level.

### Anthropometric measurements in the newborn and an AD diagnosis by age 3 years

Increasing birth length was positively associated with an AD diagnosis by age 3 years (aOR = 1.06 [95% CI = 1.01-1.12]), corresponding to a 6% increase in odds per increasing centimeter of birth length (Tables IV and V). Short birth length was associated with reduced likelihood of being diagnosed with AD by age 3 years (aOR = 0.71 [95% CI = 0.51-1.00]) (Fig 2, *B*).

**Table IV:** Overview of the population of the present study from the PreventADALL mother-child birth cohort comparing newborn anthropometric measurements and AD at age 3 years

Characteristics	AD by age 3 y			*P* value
	No	Yes	Total	
Subjects (n = 2105), no. (%)	1513 (75)	502 (25)	2015 (100)	
Birth weight (g) (n = 2031), mean ± SD	3564.4 ± 481.2	3596.9 ± 448.7	3572.5 ± 473.5	.181
Low (<2994 g) (n = 203), no. (%)	163 (80)	40 (20)	203 (100)	
Normal (2994-4180 g) (n = 1626), no. (%)	1210 (74)	416 (26)	1626 (100)	
High (>4180 g) (n = 206), no. (%)	156 (76)	50 (24)	206 (100)	
Birth length (cm) (n = 1937), mean ± SD	50.4 ± 2.0	50.7 ± 2.0	50.5 ± 2.0	.007
Low (<48 cm) (n = 280), no. (%)	226 (81)	54 (19)	280 (100)	
Normal (48-53 cm) (n = 1529), no. (%)	1132 (74)	397 (26)	1529 (100)	
High (>53 cm) (n = 128), no. (%)	93 (73)	35 (27)	128 (100)	
Upper arm circumference (n = 2031), mean ± SD	11.2 ± 1.0	11.2 ± 1.0	11.2 ± 1.0	.717
Low (<10 cm) (n = 248), no. (%)	199 (80)	49 (20)	248 (100)	
Normal (10-12.4 cm) (n = 1600), no. (%)	1196 (75)	404 (25)	1600 (100)	
High (>12.4 cm) (n = 183), no. (%)	131 (72)	52 (28)	183 (100)	
Thoracic circumference (n = 2024), mean ± SD	34.0 ± 1.9	34.1 ± 1.8	34.0 ± 1.9	.356
Low (<31.7 cm) (n = 221), no. (%)	171 (77)	50 (23)	221 (100)	
Normal (31.7 -36.4 cm) (n = 1624), no. (%)	1219 (75)	405 (25)	1624 (100)	
High (>36.4 cm) (n = 179), no. (%)	131 (73)	48 (27)	179 (100)	
Abdominal circumference (n = 2023), mean ± SD	32.8 ± 2.2	32.8 ± 2.1	32.8 ± 2.2	.708
Low (<30 cm) (n = 222), no. (%)	165 (74)	57 (26)	222 (100)	
Normal (30-35.5 cm) (n = 1614), no. (%)	1219 (76)	395 (24)	1614 (100)	
High (>35.5 cm) (n = 187), no. (%)	136 (73)	51 (27)	187 (100)	
Abdominal-to-thoracic ratio (n = 2020), mean ± SD	1.0 ± 0.0	1.0 ± 0.0	1.0 ± 0.0	.496
Low (<0.91) (n = 164), no. (%)	129 (79)	35 (21)	164 (100)	
Normal (0.91-1.02) (n = 1678), no. (%)	1248 (74)	430 (26)	1678 (100)	
High (>1.02) (n = 178), no. (%)	140 (79)	38 (21)	178 (100)

**Table V:** Logistic regression of newborn anthropometric measurements (birth weight; birth length; and upper arm, abdominal, and thoracic circumference) and the risk of AD in children by age 3 years, unadjusted and adjusted for relevant confounders

Characteristics	Crude OR (95% CI)∗	*P* value∗	aOR (95% CI)†	*P* value†
Birth weight (g) (n = 2031), mean (range)	1.00 (0.99-1.00)	.181	1.00 (0.99-1.00)	.460
Low (<2994 g) (n = 203)	0.71 (0.50-1.03)	.069	0.80 (0.55-1.17)	.244
Normal (2994-4180 g) (n = 1626)	1		1	
High (>4180 g) (n = 206)	0.93 (0.67-1.31)	.684	0.95 (0.66-1.35)	.763
Birth length (cm) (n = 1937), mean (range)	1.07 (1.02-1.13)	.007	1.06 (1.01-1.12)	.028
Low (<48 cm) (n = 280)	0.68 (0.50-0.94)	.018	0.71 (0.51-1.00)	.048
Normal (48-53 cm) (n = 1529)	1		1	
High (>53 cm) (n = 128)	1.07 (0.72-1.61)	.733	1.02 (0.67-1.57)	.925
Upper arm circumference (n = 2031), mean (range)	1.02 (0.92-1.13)	.716	0.99 (0.90-1.11)	.988
Low (<10.0 cm) (n = 248)	0.73 (0.52-1.02)	.062	0.76 (0.54-1.08)	.127
Normal (10.0-12.4 cm) (n = 1600)	1		1	
High (>12.4 cm) (n = 183)	1.18 (0.84-1.65)	.353	0.99 (0.68-1.44)	.952
Thoracic circumference (n = 2024), mean (range)	1.03 (0.97-1.08)	.356	1.02 (0.96-1.08)	.545
Low (<31.7 cm) (n = 221)	0.88 (0.63-1.23)	.454	0.97 (0.69-1.37)	.857
Normal (31.7-36-4 cm) (n = 1624)	1		1	
High (>36.4 cm) (n = 179)	1.10 (0.78-1.56)	.583	1.06 (0.73-1.54)	.755
Abdominal circumference (n = 2023), mean (range)	1.01 (0.96-1.06)	.708	0.99 (0.95-1.05)	.821
Low (<30.0 cm) (n = 222)	1.07 (0.77-1.47)	.697	1.19 (0.85-1.67)	.316
Normal (30.0-35.5 cm) (n = 1614)	1		1	
High (>35.5 cm) (n = 187)	1.16 (0.82-1.63)	.401	1.14 (0.79-1-65)	.474
Abdominal-to-thoracic ratio (n = 2020), mean (range)	0.43 (0.039-4.82)	.495	0.23 (0.018-2.90)	.253
Low (<0.91) (n = 164)	0.79 (0.53-1.16)	.229	0.85 (0.56-1.28)	.438
Normal (0.91-1.02) (n = 1678)	1		1	
High (>1.02) (n = 178)	0.79 (0.54-1.15)	.212	0.80 (0.54-1.18)	.257

When categoric variables were applied, the 10th, 50th, and 90th percentiles were used as thresholds.

*OR*, Odds ratio.

∗ ORs and *P* values from univariable logistic regression models investigating the association between newborn anthropometric measurements and the risk of AD by age 3 years.

† ORs and *P* values from multivariable logistic regression models investigating the association between newborn anthropometric measurements the risk of AD by age 3 years, adjusted for maternal education and maternal ppBMI.

Neither birth weight nor circumferences of the upper arm, thorax, or abdomen at birth were associated with an AD diagnosis by age 3 years (Fig 2, *C*).

### Fetal growth and AD by age 3 years

Although, increasing fetal weight gain was associated with an increased risk of AD development by age 3 years in the univariable analyses (OR = 1.08 [95% CI = 1.00-1.15]), the association did not remain statistically significant after adjustment (aOR = 1.07 [95% = CI 0.99-1.15]) (Table VI). Likewise, a nonsignificant tendency for higher odds of AD in children was observed with increasing fetal thoracic circumference growth (aOR =11.31 [95% CI 0.78-163.1]; *P* = .075). Fetal abdominal circumference growth, as well as and low and high growth rates, were not associated with AD (Table VI).

**Table VI:** Logistic regression of fetal growth (fetal weight gain, fetal thoracic, and abdominal circumference) and the risk of AD in children by age 3 years of age, unadjusted and adjusted for relevant confounders

Characteristics	Crude OR (95% CI)∗	*P* value∗	aOR (95% CI)†	*P* value†
Fetal weight gain (mg/d) (n = 1,578), mean (range)	1.08 (1.00-1.15)	.043	1.07 (0.99-1.15)	.098
Low (<10,650 mg) (n = 160)	0.79 (0.54-1.17)	.242	0.89 (0.59-1.35)	.594
Normal (10,650-14,640 mg) (n = 1262)	1		1	
High (>14,640 mg) (n = 156)	1.00 (0.69-1.46)	.986	1.05 (0.71-1.57)	.798
Fetal thoracic circumference growth (mm/d) (n = 591), mean (range)	6.71 (0.56-80.50)	.133	11.31 (0.78-163.10)	.075
Low (<0.75) (n = 59)	0.93 (0.50-1.72)	.805	0.90 (0.47-1.72)	.746
Normal (0.75-0.94) (n = 479)	1		1	
High (>0.94) (n = 53)	1.40 (0.76-2.55)	.279	1.39 (0.73-2.66)	.319
Fetal abdominal circumference growth (mm/d) (n = 717), mean (range)	1.27 (0.17-9.25)	.814	1.12 (0.14-8.97)	.913
Low (<0.62) (n = 73)	1.18 (0.69-2.01)	.543	1.13 (0.64-1.99)	.670
Normal (0.62-0.83) (n = 579)	1		1	
High (>0.83) (n = 65)	1.30 (0.75-2.27)	.343	1.35 (0.77-2.38)	.294

Fetal growth, including fetal weight gain and fetal thoracic and abdominal circumference growth, are continuous variables, and the rest are categoric.

*OR,* Odds ratio.

∗ ORs and *P* values from univariable logistic regression models investigating the association between fetal growth and the risk of AD by age 3 years.

† ORs and *P* values from multivariable logistic regression models investigating the association between fetal growth and the risk of AD by age 3 years, adjusted for maternal ppBMI and maternal educational level.

## Discussion

In the present study, we observed that increasing maternal ppBMI was significantly associated with an increased risk of AD in the mother’s child by age 3 years. Although not statistically significant, when ppBMI was categorized according to the WHO classification, there was a trend of less offspring AD among underweight mothers and more offspring AD among overweight and obese mothers. Short birth length was associated with lower risk of AD, whereas increasing birth length was associated with increased risk of AD development by age 3 years. Birth weight, thoracic, abdominal or upper arm circumference, and fetal growth were not associated with AD development by age 3 years.

Our finding that increasing maternal ppBMI is associated with offspring AD by age 3 years is in conflict with the findings of a prospective cohort study of US children with 13,269 participants, 2,058 of whom (16%) had childhood AD; in addition, no association was observed between maternal ppBMI and child with AD aged 10 to 17 years.^8^ The US study reported increasing maternal GWG as being associated with increased AD risk.^8^ Although we observed an association between increasing maternal ppBMI and offspring AD, the effect size was small, and because the CI included a value of 1.0, the association is uncertain and unlikely to be of clear clinical relevance. A study from Japan that included 67,204 mother-child pairs also found no association between ppBMI and AD in the child at age 3 years.^22^ Our results are also in conflict with the findings of a systematic review and meta-analysis of 114,485 participants^9^ that covered 10 studies on ppBMI and 5 on maternal GWG in relation to offspring AD between age 6 months and age 17 years. The authors of that study reported an association between maternal underweight as well as moderate to very high maternal GWG and increased risk of childhood AD, whereas maternal ppBMI as a continuous variable was not associated with childhood AD.^9^

Several methodologic differences may explain these discrepancies. In both the US and Japanese cohorts, AD was identified by using parental questionnaires and self-reported diagnoses, whereas our study utilized clinical assessment based on validated diagnostic criteria at multiple timepoints. This difference in case ascertainment may have influenced sensitivity and specificity. Additionally, age at AD evaluation varied, with our study and the Japanese cohort focusing on children by age 3 years whereas the US study included older children and adolescents, which may have affected diagnostic precision.

Our observation that increased birth length is associated with a higher risk of AD by age 3 years whereas shorter birth length appears to reduce this risk stands in contrast to the findings from the Ulm SPATZ Health Study Cohort,^10^ which included 1006 live newborns of 970 mothers recruited soon after delivery at the University Medical Centre Ulm in Germany in 2012-2013.^10^ The Ulm SPATZ Health Study examined the association between trimester-specific ultrasound-based anthropometric measurements and the diagnosis of AD by age 3 years.^10^ Specifically, the authors assessed first-trimester crown-to-rump length, second- and third-trimester head circumference, abdominal circumference, femur length, and EFW by using the Hadlock 3 formula, as well as trimester-specific ratios of head circumference to abdominal circumference. The study found that among the 1006 newborns, impaired fetal growth in the first trimester may be linked to an increased risk of AD development, whereas greater body length in the third trimester could be protective against AD development by age 3 years.^10^ The diagnosis of AD in this cohort was based on parent and physician reports, and in some cases, dermatologic examination records, according to which 197 children (26.8%) were diagnosed with AD by age 3 years.^10^ In our study, although the association between birth length and AD was statistically significant, the effect size was small and the CI was close to 1.0, suggesting limited clinical relevance.

Our finding that birth weight was not associated with an increased risk of AD contrasts with the findings from a prospective multicenter cohort study that was conducted in Italy and included 1081 participants. In that study, higher birth weight was modestly associated with an increased risk of AD at ages 6 and 12 months, as determined by questionnaire-based assessments.^23^

The observed association between increased birth length, but not birth weight, and a higher risk of AD by age 3 years warrants further consideration. During pregnancy, linear growth is influenced by growth hormone and insulin-like growth factor signaling, which may also play roles in immune system development and skin barrier maturation.^24^ It is possible that exposures or genetic factors promoting greater linear growth *in utero* could simultaneously affect pathways predisposing to AD. Alternatively, birth length may serve as a proxy for other influences, such as parental height, placental function, or maternal nutrition, which could confound the observed association. With respect to postnatal linear growth, a systematic review examining 14 studies with a total of 50,146 patients with AD found no evidence of an association with risk of AD development.^25^ Although our analyses adjusted for maternal education and ppBMI, residual confounding cannot be excluded. More research is needed to understand why this relationship exists, as well as to find out whether birth length may add to an increased risk of AD or whether it simply reflects other risk factors.

To our knowledge, this is the first study to identify a possible association between increased fetal weight gain and the development of AD by age 3 years. Although the results were no longer significant with adjustment for confounders, they are in contrast with the findings of the Ulm SPATZ Health Study, which reported that impairment of early fetal growth during the first trimester may increase risk of AD.^10^

We did not observe a significant association between fetal abdominal or thoracic growth and the risk of AD development by age 3 years. This finding contrasts with the results of the Ulm SPATZ Health Study, which reported that both low and high second-trimester abdominal circumference and an elevated ratio of head circumference to abdominal circumference in the second trimester were associated with an increased risk of AD.^10^ To our knowledge, aside from the present analysis, no other studies have specifically investigated fetal thoracic circumference in relation to AD development. However, a prospective longitudinal pregnancy cohort study at the University Medical Centre Hamburg-Eppendorf that included 177 children found that fetal lung growth made it possible to predict the risk of asthma at age 5 years.^26^ Both our study and the Ulm SPATZ Health Study identified specific patterns of fetal growth that may influence the risk of AD development, possibly owing to similar population characteristics in the respective countries (or populations). The differences in results between the studies could be due to variations in ultrasound measurement techniques or timing. Additionally, a Swedish twin study including 11,020 twins found a positive association between high fetal growth and childhood AD.^27^

Maternal overweight may influence the risk of offspring AD through several mechanisms. Higher maternal BMI is associated with an increased prevalence of caesarean delivery, and caesarean delivery has in turn been linked to a higher risk of childhood AD.^28^^,^^29^ Mothers with prepregnancy overweight or obesity exhibit molecular alterations in the skin barrier and staphylococcal dysbiosis, both of which can lead to a proinflammatory state and increased risk of AD development.^30^

Our findings should be interpreted in the context of a complex and sometimes contradictory literature. Although several meta-analyses and cohort studies have reported that maternal underweight or excessive GWG may increase the risk of childhood AD, the association with maternal BMI as a continuous variable remains inconsistent across studies.^8^^,^^9^ Similarly, the evidence for early anthropometric measurements such as birth length or weight is mixed, with some studies suggesting an influence on AD risk.^10^ Maternal adiposity may affect fetal immune and skin barrier development through proinflammatory effects.^30^ It is also possible that unmeasured genetic, familial, or environmental factors contribute to the observed associations. Thus, further research is needed to clarify these relationships and underlying mechanisms

The PreventADALL cohort benefits from several strengths, including a large, unselected pregnancy population, long-term offspring follow-up, and a stringent skin assessment by trained personnel. The use of validated diagnostic tools enhances the reliability of findings compared with that of the findings of other studies reliant on questionnaires and retrospective data. However, the predominantly Scandinavian participants limit the generalizability, and relatively few individuals in extreme anthropometric subgroups may reduce statistical power. Another potential limitation relates to selection bias. Although a substantial proportion of participants were excluded owing to missing exposure or outcome data, which could potentially have introduced selection bias, we compared key baseline characteristics between the included and excluded participants. The distributions were similar, suggesting that major systematic differences are unlikely and that the risk of selection bias is probably limited. The participants were randomly selected and included from a general population, which is also reflected in the baseline characteristics; however, the frequency of maternal atopic disease was slightly higher than in other Scandinavian general population studies, which may have introduced a selection bias, but should have been taken care of when adjusting for maternal AD.

In addition, some measurement error in obtaining fetal ultrasound measurements cannot be ruled out. Fetal weight gain was calculated using EFW obtained by ultrasound at midpregnancy and birth weight at delivery, and combining estimated and measured weights may introduce measurement error due to differences in precision and timing of the assessments.

### Conclusion

Our study of around 2000 mother-child pairs showed that increasing maternal ppBMI and increasing birth length were associated with higher risk of AD development in children by age 3 years. In contrast, short birth length appeared to be associated with reduced risk. Our results highlight the importance of early-life growth patterns for the development of AD.

## Disclosure statement

The PreventADALL study has received funding from the following sources, none of which had any influence on the design, conduct, or analyses of the PreventADALL study: the Regional Health Board South East; the Norwegian Research Council; Oslo University Hospital; the University of Oslo; Health and Rehabilitation Norway; the Foundation for Healthcare and Allergy Research in Sweden–Vårdalstiftelsen; the Swedish Asthma and Allergy Association’s Research Foundation; the Swedish Research Council–the Initiative for Clinical Therapy Research; the Swedish Heart-Lung Foundation; SFO-V Karolinska Institutet; Østfold Hospital Trust (by unrestricted grants from the Norwegian Association of Asthma and Allergy); the Kloster foundation; Thermo-Fisher, Uppsala, Sweden (in the form of provision of allergen reagents); Fürst Medical Laboratory, Oslo, Norway (in the form of conductiing IgE analyses); the Norwegian Society of Dermatology and Venerology; Arne Ingel’s legat; Region Stockholm (through the ALF project and individual grants); Forte, Swedish Order of Freemasons Foundation Barnhuset; the Sven Jerring Foundation; the Hesselman Foundation; the Magnus Bergwall Foundation; the Konsul Th. C. Bergh’s Foundation; the Swedish Society of Medicine, the King Gustaf V 80th Birthday Foundation; Karolinska Institutet grants; the Cancer and Allergy Foundation; the Pediatric Research Foundation at Astrid Lindgren Children’s Hospital; the Samaritan Foundation for Pediatric Research; the Barnestiftelsen at Oslo University Hospital; Roche; and the Frithjof Nansen Institute.

Disclosure of potential conflict of interest: The authors declare that they have no relevant conflicts of interest.
