# Supplementary material for: Maternal BMI, early-life growth, and atopic dermatitis by age 3 years
Source: J Allergy Clin Immunol Glob. 2026 Mar 23;5(3):100693. doi: 10.1016/j.jacig.2026.100693 (PMC13091986; doi:10.1016/j.jacig.2026.100693)
Supplement: Supplementary Material [file mmc1.docx]

**Fig 1. Directed Acyclic Graph for aim 1: whether maternal pre-pregnancy overweight is associated with offspring AD by three years of age. The identified confounders were mothers educational level and maternal AD.**


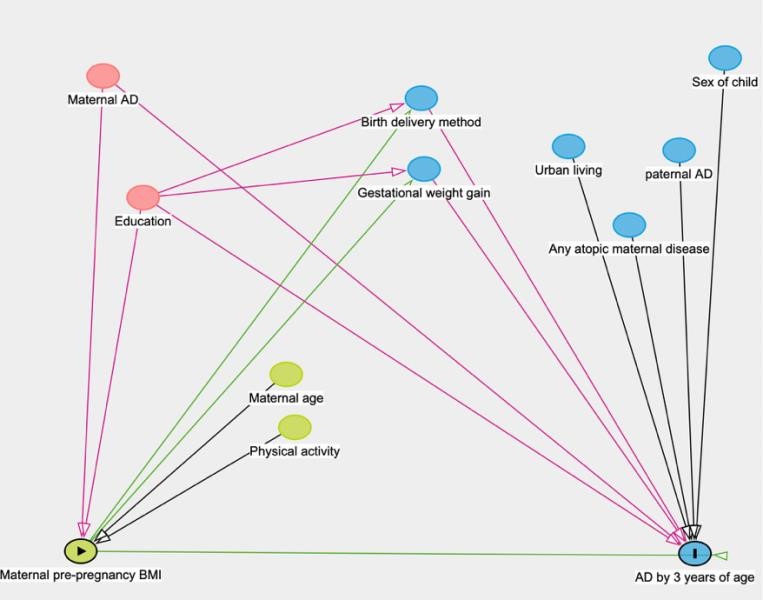


**Fig 2. Directed Acyclic Graph for aim 2: whether anthropometric measurements in the newborn are associated to the development of AD by three years of age. The identified confounders were mothers educational level and maternal pre-pregnancy BMI.**


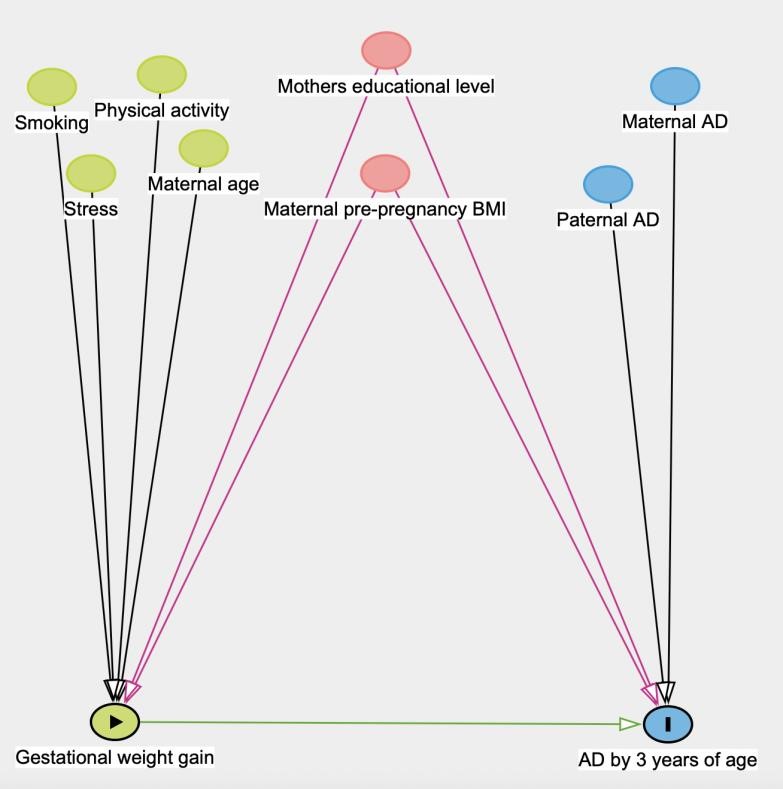


**Fig 3. Directed Acyclic Graph for aim 3: whether fetal weight gain, as well as if fetal thoracal and abdominal circumference growth are associated with AD by three years of age. The identified confounders were mothers educational level and maternal pre-pregnancy BMI.**


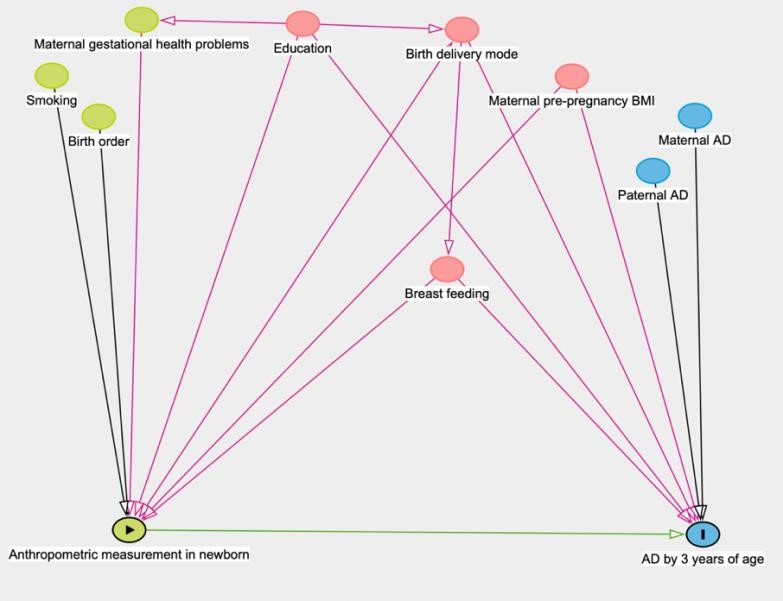


**TABLE 1 Baseline characteristics of study population of the present study, from the Preventing Atopic Dermatitis and Allergies in children (PreventADALL) mother-child birth cohort comparing those in this study population versus those who are not included in the study population**

| Characteristics | Included (n= 2107) | Not included (n= 276) | Total (n= 2383) P-value |
| --- | --- | --- | --- |
| No. of subjects | 2107 (88) | 276 (12) | 2383 (100) |
| Sex (n=2383) |  |  | **0.022** |
| Female | 983 (46) | 149 (54) | 1132 (48) |
| Male | 1124 (54) | 127 (46) | 1251 (52) |
| Dwelling (n=2160) |  |  | **0.038** |
| City | 1482 (70) | 177 (64) | 1659 (70) |
| Suburb | 306 (15) | 38 (14) | 344 (14) |
| Countryside | 131 (6) | 26 (9) | 157 (7) |
| Education (n=2151) |  |  | **0.000** |
| Pre/high school | 197 (9) | 40 (15) | 237 (10) |
| Higher education < 4y | 592 (28) | 94 (34) | 686 (29) |
| Higher education => 4y | 1121 (53) | 107 (39) | 1228 (52) |
| Mother origin (n=2160) |  |  | 0.659 |
| Scandinavia | 1739 (83) | 213 (77) | 1952 (82) |
| Rest of the world | 180 (9) | 28 (10) | 208 (9) |
| Mother diagnosed with AD (n= 2160) |  |  | 0.982 |
| Yes | 381 (18) | 48 (17) | 429 (18) |
| No | 1538 (73) | 193 (70) | 1731 (73) |
| Mother diagnosed with asthma (n=2160) |  |  | 0.168 |
| Yes | 322 (15) | 49 (18) | 371 (16) |
| No | 1597 (76) | 192 (70) | 1789 (75) |
| Mother diagnosed with any atopic diseases (n=2160) |  |  | 0.228 |
| Yes | 790 (38) | 109 (40) | 899 (38) |
| No | 1129 (54) | 132 (48) | 1261 (53) |
| Filaggrin mutation in child (n=2383) |  |  | **0.000** |
| Yes | 150 (7) | 16 (6) | 166 (7) |
| No | 1505 (71) | 159 (58) | 1664 (70) |
| Unknown | 452 (22) | 101 (37) | 553 (23) |
| Birth mode (n= 2380) |  |  | 0.253 |
| Vaginal | 1771 (84) | 218 (79) | 1989 (84) |
| C- section | 334 (16) | 57 (21) | 391 (16) |
| Age of mother (years) (n=2383) | 32.5 [4.12] | 31.8 [4.30] | 32.4 [4.15] **0.011** |
| GA at birth (weeks) (n=2377) | 40.06 [9.44] | 40.04 [8.90] | 40.06 [9.38] 0.746 |
| Birth weight (g) (n=2374) | 3573.51 [476.21] | 3588.16 [486.44] | 3575.20 [477.32] 0.633 |

The once marked with * means it is a continuous variable, and the rest are categorical variables. Categorical variables are presented as numbers and percentages, while continuous variables are reported as means, standard deviations (SD), and minimum (min) to maximum (max) values.

**TABLE 2 Overwiew of the study population of the present study, from the Preventing Atopic Dermatitis and Allergies in children (PreventADALL) mother-child birth cohort comparing pre-pregnancy BMI and AD by 36 months**

**AD in infant by 36 months**

| Characteristics | No | Yes | Total P-value |
| --- | --- | --- | --- |
| No. of subjects (n=2107) | 1582 (75) | 525 (25) | 2107 (100) |

| Maternal pre-pregnancy BMI (kg/m^2) (n=2103) | 24.689 [3.63] | 24.971 [3.64] | 24.759 [3.6] 0.125 |
| --- | --- | --- | --- |
| Underweight (<18.5) (n=31) | 26 (84) | 5 (16) | 31 (100) |
| Normal weight (18.5-24.9) (n=1637) | 1231 (75) | 406 (25) | 1637 (100) |
| Overweight (25.0-29.9) (n=326) | 239 (73) | 87 (27) | 326 (100) |
| Obese (>30) (n=113) | 86 (76) | 27 (24) | 113 (100) |

The once marked with * means it is a continuous variable, and the rest are categorical variables. Categorical variables are presented as numbers and percentages, while continuous variables are reported as means, standard deviations (SD), and minimum (min) to maximum (max) values.

| Standard Operating Procedure: Anthropometrics NEWBORN | | | | 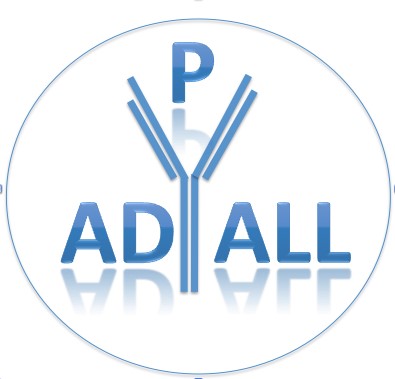 |
| --- | --- | --- | --- | --- |
|  | Rev. No.   1. (11.05.15) 2. (31.07.15) 3. (11.11.15) | Date: 28.04.2015 | Page **1** of **2** |  |

Purpose:

Recording anthropometric measurements of child to assess growth

Possible interferences:

Uneasy child

Medical conditions in child preventing proper measurements

Equipment:

Tape measurer

Skin marker

Surface disinfectant

Personnel qualifications:

Trained health professional

Safety considerations:

Disinfectant on tape measure before and after every examination

Procedure:

Inform the parents about the procedure and ask for their permission to make a mark with the skin marker on the shoulder, the elbow and in the middle of the upper arm.

# Left upper arm circumference

1. With the left elbow joint in a 90 degrees angle and the humerus in a vertical line perpendicular to the torsoe, mark (with a skin marker) the acromial and olecranon process and measure the length of the upper arm in a straight line once. Register the distance and make a mark halfway.


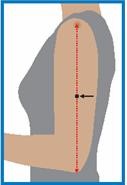

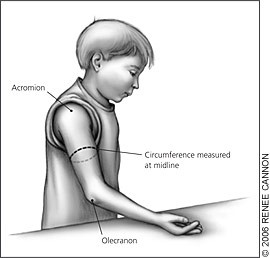


1. Place the centre of the tape measure on the mark halfway on the upper arm, and while holding the elbow in a 90 degrees angle measure the circumference. Do not tighten the tape measurer while measuring.
2. Measure and register the circumference of the left upper arm twice.

[**http://www.aafp.org/afp/2006/0501/p1558.html (**](http://www.aafp.org/afp/2006/0501/p1558.html)**26.4.15)** [**http://www.bradford-dietetics.org/accessing-our-hospital-basedservices/must (**](http://www.bradford-dietetics.org/accessing-our-hospital-based-services/must)**26.04.15)**

1

| Standard Operating Procedure: Anthropometrics NEWBORN | | |  | 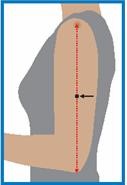 |
| --- | --- | --- | --- | --- |
|  | Rev. No. | Date: 28.04.2015 | Page **2** of **2** |  |

#
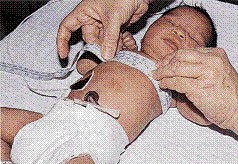
Thorax circumference

1. Place the tape measure around thorax so that the lower part of the tape measure is in line with the most caudal part of the xyphoid process. Do not tighten the tape measure while recording the circumference.
2. For newborns, leave the hip joint in a flexed position.
3. Measure and register circumference three times.

http://intranet.tdmu.edu.ua/data/kafedra/internal/ginecology2/classes_stud/en/nurse/bsn/ptn/4/Nursing%20Care%20of%20Childbearing%20Family_Practicum/2 3.%20Newborn%20assessment.files/image025.gif

#
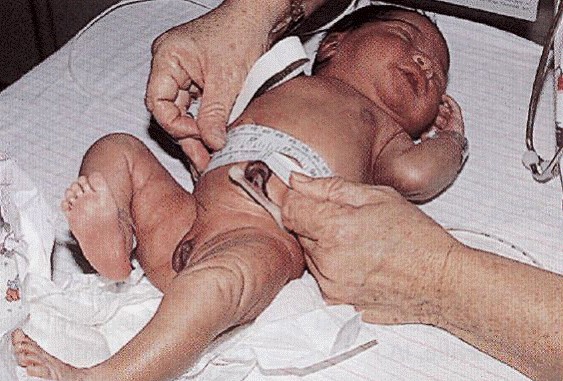
Abdominal circumference

1. Place the tape measure around the abdomen so that the lower part of the tape measure is in line with the cranial part of the navel. Do not tighten the tape measure while recoding the circumference.
2. For newborns, let the hip joint in a flexed position.
3. Measure and register circumference three times.

http://intranet.tdmu.edu.ua/data/kafedra/internal/ginecology2/classes_stud/en/nurse/bsn/ptn/4/Nursing%20Care%20of%20Childbearing%20Family_Practicum/2 3.%20Newborn%20assessment.files/image026.gif

2

Purpose:

The purpose is to obtain information about fetal biometric parameters, pregnancy length and to make a survey of the fetal anatomy, amniotic fluid volume, and placenta or pregnancy pathology.

Possible interferences:

Equipment:

Ultrasound machine with software suitable for performing obstetric ultrasound.

Voluson Pro, Expert, E6 or E8; all General Electrics

Personnel qualifications:

Health personnel (midwife) trained in performing ultrasound screening in pregnancy.

Safety considerations:

The performer should follow the general principle concerning energy transmission to the fetus. “the ALARA principle” (as low as reasonable achievable).

Procedure:

The ultrasound operator takes the obstetric history and performs ultrasound examination of the foetus, placenta and amniotic fluid. A short organ scan and foetal biometry is done. The due date for delivery is estimated according to the local guidelines.

The following parameters need to be recorded:

1. Biparietal diameter (BPD) (in millimetres with one decimal): average of three measurements
2. Head circumference (HC) (in millimetres with one decimal): average of three measurements
3. Abdominal circumference (AC) (in millimetres with one decimal): average of three measurements
4. Femur length(FL) (in millimetres with one decimal): average of three measurements
5. Thoracic circumference (TC)(in millimetres with one decimal): average of three measurements. An ellipse is placed around the bony structures of the thorax in the axial plane at the level of the four chamber view of the heart. See picture.
6. The largest amniotic fluid pocket (AF) (in centimetres with one decimal): The largest anteriorposterior diameter of the largest amniotic fluid pocket.
7. Placental position.
8. Any structural foetal anomalies observed

The Routine scan form is filled out and three pictures of thoracic circumference measurement are attached to the form (see form: data registrert ved ultralyd).

All, except TC, AF and in some clinics BDP are routine examinations.


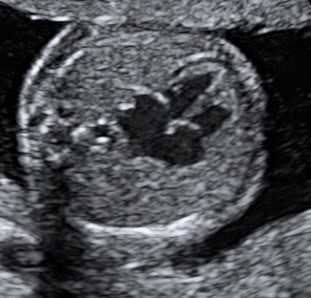


## Reference values (mm) for routine scan Biparietal diameter (BPD)

| **Pregnancy week** | **5^th^ centile** | **50^th^ centile** | **95^th^ centile** |
| --- | --- | --- | --- |
| **17** | 34 | 38 | 43 |
| **18** | 38 | 42 | 46 |
| **19** | 41 | 45 | 50 |
| **20** | 44 | 49 | 54 |

## Head circumference (HC)

| **Pregnancy week** | **5^th^ centile** | **50^th^ centile** | **95^th^ centile** |
| --- | --- | --- | --- |
| **17** | 121 | 134 | 149 |
| **18** | 133 | 147 | 162 |
| **19** | 145 | 159 | 175 |
| **20** | 157 | 172 | 187 |

## Abdominal circumference (AC)

| **Pregnancy week** | **5^th^ centile** | **50^th^ centile** | **95^th^ centile** |
| --- | --- | --- | --- |
| **17** | 102 | 116 | 132 |
| **18** | 113 | 128 | 144 |
| **19** | 124 | 140 | 157 |
| **20** | 135 | 151 | 169 |

## Femur length (FL)

| **Pregnancy week** | **5^th^ centile** | **50^th^ centile** | **95^th^ centile** |
| --- | --- | --- | --- |
| **17** | 18 | 21 | 26 |
| **18** | 21 | 25 | 29 |
| **19** | 24 | 28 | 33 |
| **20** | 27 | 31 | 36 |

## Thoracic circumference (TC) *

| **Pregnancy week** | **5^th^ centile** | **50^th^ centile** | **95^th^ centile** |
| --- | --- | --- | --- |
| **17** | 73 | 100 | 118 |
| **18** | 82 | 110 | 129 |
| **19** | 91 | 119 | 146 |
| **20** | 100 | 128 | 155 |

*S.L. Johnsen et al., EJOGRB, 2006; 127: 172 -185* (for BPD, HC, AC, FL)

*Chitkara et al., AJOG, 1987; 156: 1069-74* (for TC) * Reference values may be slightly higher due to the method of measurement .
